# Dystokia1Read before the meeting of the Pennsylvania State Veterinary Medical Association, March 5, 1901.

**Published:** 1901-07

**Authors:** J. Curtis Michener

**Affiliations:** Colmar, Pa.


### DYSTOKIA.[1]

By J. Curtis Michener, V.S.,
COLMAR, PA.

To such of us as practice in dairy and breeding districts this is a most important subject. Added to the natural sympathy we have for a creature in the maternal throes, when the labor pains are protracted and in vain, imperilling the lives of both mother and offspring, it becomes a most urgent, important, and interesting case.

Our subjects are called dumb brutes. Who can witness the very anxious expression, the awful agonizing efforts made in dystokial cases, and the evident rejoicing and fondness displayed by the dam when delivery is effected, without recognizing the consciousness she has of what is going on, and having our manhood stirred and arm nerved for Herculean efforts in such cases? Yes, Brother Veterinarians, I pride myself upon and deem these the most important we meet in practice. In cases of sickness our prescriptions are ofttimes of doubtful utility, but in these instances we can be positive of having saved life and property.

The subject would fill a large volume, and in a mere paper upon such an occasion details must be omitted and only some of the leading principles outlined. Anatomical and physiological knowledge is of primary importance. Unless one knows the location, texture, and functions of the reproductive organs and their relations, and is perfectly acquainted with the act of normal parturition at all of its stages, by both sight and touch, he is ill prepared to correct the manifold deviations, abnormal conditions, malformations, and misrepresentations we meet. Indeed, the abnormal conditions are so various that a great many different procedures are necessary to successfully meet the individual cases. Careful examination and cool deliberation are the first requisites, and the opposite of much practice I have seen, where without attention to the maternal condition and position, or even fully making out the position of the fœtus, or whether the inclination of the uterus favors the expulsion of its contents, or the vagina its passage, a man start in and then try to deliver by force alone. First correct everything that is wrong, then our work is done, unless the animal is worn out by futile efforts, or the actual conformations of parts will not admit of delivery, when we have a case for embryotomy or gastro-

[1] Read before the meeting of the Pennsylvania State Veterinary Medical Association, March 5, 1901.

hysterectomy. There are two points in this connection which I wish to emphasize in hope my remarks may benefit someone. The first is that the uteri of quadrupeds is a suspended bag, supported by the broad ligaments, and as it becomes filled can swing to and fro like a hammock, and whirl completely over in violent movements of the body, and dip forward or backward as the weight of the fœtus is thrown in these directions, and the position of the maternal body favors the inclination. In some cases where causes operate for long periods the uterus is displaced ; contractions and corresponding elongations in the suspending ligaments until the organ has a permanent deflection, or twist, or may have sunk so far below the pelvic passage that the young creature can never mount the precipice without help. Uneven floors, holes, and gutters under the hind feet and those low both front and back, throwing weight of body upon the soft parts, are potent causes. These and other causes too numerous to mention make work for us.

The next point I wish to make is that we go at it like rational beings. In nearly all cases where the fœtus does not present properly the uterus is somewhat distorted, putting the cervix and os uteri more or less of a twist, with a partial fold of mucous membrane formed in the vagina. The wedge-shaped head or thighs have not entered the passage to dilate it, and when we are called, after several hours of labor and rough treatment, and just plunge in to correct the displacement and extract the young one, by the rules of the books, we have a mighty dry, tough time of it. How it does squeeze the arm and take the nerve out of a fellow. The boy that does that kind of business (in plain language) is a fool. It took me several years to find it out. Since that I have found other older boys, hence the choice of this subject.

Now I mean to give you the key. All deflections of the womb from the lateral to the vertical, from partial to complete torsion, *can be remedied by changing the maternal position.* After this is effected the dry, tumefied parts can be relaxed and mollified by copious injections of warm lobelia tea, soapsuds and glycerin, and you can then accomplish a heretofore almost impossible task with an ease that will make you laugh.

As these ideas may be new to some of you, I will give mode of procedure in a few cases. I use the old-fashioned English casting straps, rope and pulleys, a lot of bags filled with bran or other light material, warm water by the bucketful, Castile soap, glycerin, a large funnel with neck bent at an acute angle inserted into two feet of gum tubing, and plenty of help. Better take the obstetrical tools along for the sake of scientific appearances ; some might come handy.

Suppose we have an anterior presentation, feet appearing out of vulva, head turned back to the flank, nose upward, been in labor until parts are dry, uterus contracted, holding the fœtus in vise-like grip. Order your warm soapsuds got ready, pulleys up over hindquarters, front legs bent at the knees and fastened thus by quiller straps around arms and pasterns, buckle your straps around hind canons, well wrapped, hook your pulley into the rings of both straps, turn up the back opposite way to which fœtal head is turned, moderate traction upon pulley rope, men lifting with blanket under hind quarters, slip under the stuffed bags, when high as prudent steady and support her there, pour in your soapsuds and glycerin (at intervals for several minutes). Now you will find it easy to push the front legs (already corded back into the uterus), with knee or knees grasped push the shoulders opposite to way neck is turned ; if the head does not come around try for it, bearing in mind that the nose must go downward and from the body. If you do not succeed, roll her more to either side as seems to loosen the womb's grip. Do not give up. Make two gallons of lobelia tea from two ounces of the dried herb, strain and pour in warm. Wait a few minutes, and the relaxation will be marvellous, giving an easy chance to adjust ready for the reaction, *when away she comes* if you have pluck and gumption. If there be torsion, right it by rolling patient's body the contrary way. This, I think, illustrates the principle, to be varied, of course, to suit the case. Should the abdomen be very pendulous and fœtus wedged hard in front of the bones, put under the sling well back, buckling breeching tight, front feet bent back. Make her stand behind and kneel in front, and pray until things come right (with your help). According to the best authors, breech presentations with the feet a way forward under the body or the anterior with all of the feet engaged in the passage are hard to overcome. Flemming pictures such a case without even hinting at the easy remedy. I find such cases very simple and easy by merely standing the animal on a sharp decline. I will relate a case to illustrate, not to brag. An esteemed colleague (the holder of three veterinary diplomas) called, saying he wished my assistance, that he had worn himself completely out trying to deliver a calf, breech presentation, legs forward under the body. Found her lying head sharply up hill in the meadow. Asked if it were possible that he had been trying in that position. Yes, he knew that she ought to be on her feet, but could not make her get up. I took her by the horns, whirled her completely around, and she got up with ease. Having her held in that position until the legs were brought into the passage, I delivered a living calf in about five minutes, reminding him of an axiom that it is hard to push a load up hill, but that it will move downward of itself. By altering the position of the patient's body we can alter the presentations of the offspring, or so change its inclination that it may be readily adjusted.

